# Prognostic Impact of Serum Transthyretin and Sarcopenia on 3-Year Mortality and Respiratory-Related Hospitalizations in Idiopathic Pulmonary Fibrosis: A Prospective Cohort Study

**DOI:** 10.3390/arm94020024

**Published:** 2026-04-08

**Authors:** Akihito Okada, Akiko Nakano, Kohei Fujita, Yoshitsugu Inoue, Toshiyasu Ito, Fumitaka Hashiba, Masashi Fujikawa, Tatsuya Tanaka, Aya Mukai, Keima Ito, Yuta Mori, Kensuke Fukumitsu, Satoshi Fukuda, Yoshihiro Kanemitsu, Tomoko Tajiri, Tetsuya Oguri, Yoshiyuki Ozawa, Takayuki Murase, Hirotsugu Ohkubo

**Affiliations:** 1 Department of Respiratory Medicine, Allergy and Clinical Immunology, Nagoya City University Graduate School of Medical Sciences, 1 Kawasumi, Mizuho-cho, Mizuho-ku, Nagoya 467-8601, Japan; neverneverokada@gmail.com (A.O.); inoabove0213@yahoo.co.jp (Y.I.); ito_toshiyasu@yahoo.co.jp (T.I.); hassi1213fu@yahoo.co.jp (F.H.); fkyuji91@gmail.com (M.F.); ncu110402@gmail.com (T.T.); keimaito4869@gmail.com (K.I.); yuta_0722jp@yahoo.co.jp (Y.M.); k-fkmt@med.nagoya-cu.ac.jp (K.F.); es-fuku@med.nagoya-cu.ac.jp (S.F.); kaney32@med.nagoya-cu.ac.jp (Y.K.); tomokot@med.nagoya-cu.ac.jp (T.T.); t-oguri@med.nagoya-cu.ac.jp (T.O.); 2 Department of Respiratory Medicine and Allergy, Nagoya City University Midori Municipal Hospital, 1-77 Shiomigaoka, Midori-Ku, Nagoya 458-0037, Japan; aki_na1114@yahoo.co.jp (A.N.); il.fait.beau8931@gmail.com (A.M.); 3 Department of Respiratory Medicine, Nagoya City University East Medical Center, Nagoya 464-8547, Japan; koheifujita2026@yahoo.co.jp; 4 Department of Diagnostic Radiology, Fujita Health University School of Medicine, 1-98, Dengakugakubo, Kutsukake-cho, Toyoake 470-1192, Japan; yoshiyuki.ozawa@fujita-hu.ac.jp; 5 Department of Pathology and Molecular Diagnostics, Graduate School of Medical Sciences, Nagoya City University, 1 Kawasumi, Mizuho-cho, Mizuho-ku, Nagoya 467-8601, Japan; tmurase@med.nagoya-cu.ac.jp

**Keywords:** idiopathic pulmonary fibrosis, transthyretin, sarcopenia, mortality, hospitalization

## Abstract

**Highlights:**

**What are the main findings?**

- Lower serum transthyretin independently predicted 3-year mortality and respiratory-related hospitalization in patients with idiopathic pulmonary fibrosis, even after adjustment for disease severity.
- Sarcopenia and low appendicular skeletal muscle mass were not independent predictors of long-term outcomes.

**What are the implications of the main findings?**

- Serum transthyretin may offer complementary prognostic information reflecting systemic vulnerability in idiopathic pulmonary fibrosis.
- Incorporating biochemical nutritional markers may help improve risk assessment beyond muscle-based measures.

**Abstract:**

Background: Prognostic markers reflecting nutritional vulnerability in idiopathic pulmonary fibrosis (IPF) remain poorly defined. Methods: In this prospective cohort study, 63 stable outpatients with IPF were followed for 3 years. Sarcopenia was defined according to the 2019 Asian Working Group for Sarcopenia criteria. Serum transthyretin levels were measured concurrently. Cox proportional hazards regression, binary logistic regression, and Kaplan–Meier survival analyses were performed. Results: During follow-up, 18 patients (29%) died and 21 (33%) experienced respiratory-related hospitalization. Serum transthyretin was an independent predictor of both 3-year mortality and respiratory-related hospitalization, even after adjusting for the Gender–Age–Physiology index. Conversely, sarcopenia and low appendicular skeletal muscle mass index (ASMI) were not independently associated with either outcome. Kaplan–Meier analysis demonstrated significant differences in both mortality and hospitalization according to serum transthyretin levels. Low ASMI evaluated using sex-specific cutoffs was associated with higher mortality in the unadjusted analysis, but not with hospitalization; sarcopenia was not significantly associated with either endpoint. Conclusions: Serum transthyretin may serve as a practical biomarker of nutritional vulnerability, providing complementary prognostic information beyond muscle mass-based assessment in IPF.

## 1. Introduction

Idiopathic pulmonary fibrosis (IPF) is a progressive fibrotic lung disease associated with high mortality [1,2]. Antifibrotic therapies such as pirfenidone and nintedanib can slow down disease progression; however, they do not halt disease progression [1]. Nerandomilast, a novel therapeutic agent targeting inflammatory and fibrotic pathways, has been investigated as a potential treatment option for IPF [3]. Nevertheless, its long-term efficacy remains understudied.

IPF is characterized by reduced lung volume, impaired gas exchange, progressive dyspnea and cough, and decreased exercise capacity. The established prognostic factors include dyspnea scores [4], pulmonary function [5], the Gender–Age–Physiology (GAP) index [6], functional exercise capacity [7], and the extent of fibrosis on high-resolution computed tomography (CT) scan [8,9].

Malnutrition is an important determinant of prognosis in IPF. Nutritional status is traditionally assessed by body mass index or unintentional weight loss [10,11,12], and it can also be evaluated using serum transthyretin levels [13]. Transthyretin, formerly known as prealbumin, is a protein synthesized by the liver and has been proposed as a sensitive biomarker of nutritional status. A previous study showed that serum transthyretin is a strong predictor of 1-year risk of respiratory-related hospitalization or death in patients with IPF [14].

Sarcopenia is an age-related syndrome characterized by progressive loss of skeletal muscle mass and strength and is associated with disability, poor quality of life, and mortality [15,16]. According to the 2019 Asian Working Group for Sarcopenia (AWGS) criteria, sarcopenia is defined as low muscle mass, reduced muscle strength, or impaired physical performance [16]. Skeletal muscle loss is commonly assessed using the appendicular skeletal muscle index (ASMI), measured via dual-energy X-ray absorptiometry or bioimpedance analysis. The prevalence of sarcopenia in IPF ranges from 22.9% in Northern Italy to 39.3% in Japan [17,18]. Previous studies have examined the association between muscle mass and mortality using the fat-free mass index [19] or cross-sectional area of the erector spinae muscles on CT scan [20,21]. However, the prognostic significance of sarcopenia defined by standardized criteria remains unclear.

The current study aimed to investigate whether serum transthyretin levels and sarcopenia predict mortality and respiratory-related hospitalization in patients with IPF. Importantly, to assess their long-term prognostic significance, these associations were examined over a 3-year follow-up period. We believe that this study updates and expands the findings of previous research [14].

## 2. Materials and Methods

### 2.1. Ethics Approval and Consent to Participate

This single-center study was conducted in accordance with the Declaration of Helsinki and was approved by the Ethics Review Board of Nagoya City University Hospital (approval numbers: 60-20-0190 [24 February 2021]). A written informed consent was obtained from all participants.

### 2.2. Patients

IPF was diagnosed via multidisciplinary discussions according to the 2018 International Guidelines [2]. Between April 2021 and September 2022, 79 stable outpatients with IPF were identified and screened at Nagoya City University Hospital, Japan. The inclusion criteria included patients who provided written informed consent and were able to undergo gait speed measurement. The exclusion criterion was the presence of active cancer upon enrollment. After obtaining written informed consent, blood sampling including serum transthyretin measurement and assessment for sarcopenia were performed. The day when these evaluations were completed was defined as the beginning of the 3-year observation period.

### 2.3. Endpoint

The primary endpoint was to determine whether serum transthyretin levels and sarcopenia affect all-cause mortality within 3 years. The secondary endpoint was to examine whether serum transthyretin levels and sarcopenia affect respiratory-related hospitalization and to evaluate the association between serum transthyretin and related clinical indices, including body mass index and ASMI.

### 2.4. Sample Size

In previous reports [11], the 3-year survival in IPF was ~40% in patients with weight loss and ~80% in those without. However, these studies were conducted before or during the early phase of antifibrotic therapy use, and overall survival in IPF has improved in recent years. Considering the survival benefits associated with antifibrotic treatment, lower mortality rates were assumed in the present study. Patients with low serum transthyretin and sarcopenia were considered to represent a high-risk group with an estimated 3-year survival of ~45%, and those without ~85%. The prevalence of low serum transthyretin and sarcopenia was expected to be 35%, based on previous reports and data from our institutional cohort. Sample size was calculated based on comparison of survival curves using the log-rank test. Assuming a two-sided significance level (α) of 0.05 and a statistical power of 0.80, the required total sample size was estimated as 56 patients. Allowing for potential loss to follow-up and incomplete data, the target sample size was therefore set at 60 patients. In the absence of published mortality estimates based on this phenotype, these assumptions were considered clinically reasonable and pragmatic for sample size estimation.

### 2.5. Pulmonary Function Tests

In accordance with the American Thoracic Society/European Respiratory Society criteria, all patients underwent pulmonary function tests using a spirometer (CHESTAC-8900; CHEST M.I., Inc., Tokyo, Japan) [22]. The diffusing capacity of the lung for carbon monoxide (DL_CO_) was measured using the same spirometer. Percent predicted forced vital capacity (%FVC) and percent predicted DL_CO_ (%DL_CO_) were calculated based on the patients’ height, age, and sex according to the standardized Japanese methods [23]. The GAP index was calculated using the formula proposed by Ley et al. [6].

### 2.6. Diagnosis of Sarcopenia

In this study, sarcopenia was defined according to the AWGS 2019 consensus update [16]. Patients who had a low muscle mass and strength or poor physical performance were diagnosed with sarcopenia. Muscle mass was assessed using ASMI (kg/m^2^) and a direct segmental multi-frequency bioelectrical impedance analyzer (InBody 720; InBody Japan, Tokyo, Japan). Measurements were performed in the standing position with participants wearing light clothing. Muscle strength was evaluated using handgrip measurements obtained with an electronic dynamometer (HG-251; N-Force, Wakayama, Japan), with participants standing and elbows fully extended, as in previously reported methods [16,20]. Two trials were completed with each hand, and the highest of the four values obtained used for analysis. The cutoff criterion for low muscle strength was defined as <28.0 kg for males and <18.0 kg for females [16]. Physical performance was assessed by measuring gait speed over a 10-m corridor at the participant’s usual walking pace. Low physical performance was defined as a gait speed of <1.0 m/s for both men and women.

### 2.7. Analysis of the Erector Spinae Muscles at the Level of the 12th Thoracic Vertebra Using an Imaging Analysis Software

CT imaging analysis was performed using the SYNAPSE VINCENT software (Fujifilm Medical Systems, Tokyo, Japan) to identify the cross-sectional area of the erector spinae muscles at the level of the 12th thoracic vertebra (ESM_CSA_), which was calculated manually according to a previously published method [21]. Briefly, the ESM_CSA_ was measured on a single-slice axial CT scan image at the level of the spinous process of the 12th thoracic vertebra. For the quantitative analysis of the erector spinae muscles (ESMs), high-resolution CT scan images of the chest were reconstructed using mediastinal window settings (window level: 40 HU, window width: 300 HU). The left and right ESMs were identified and manually shaded, and the ESM area was reported as the sum of the right and left ESMs. All analyses of CT scan images were independently performed by trained individuals (FK and KI), who were blinded to the clinical information of the patients. The average ESM_CSA_ values obtained by KF (Kohei Fujita) and KI were analyzed.

### 2.8. Statistical Analyses

Continuous variables were presented as the means (±standard deviations) or medians (interquartile ranges), and categorical variables were expressed as numbers and percentages. Group differences were analyzed using Student’s t-test or the Mann–Whitney U test for continuous variables and the chi-square test for categorical variables. Univariate and multivariate Cox proportional hazards models were used to examine as-sociations between the selected variables and all-cause mortality within 3 years. To avoid model overfitting given the limited number of events, multivariable adjustment was restricted to clinically essential covariates. The primary multivariable model therefore included this phenotype and the GAP index as a measure of disease severity. Sensitivity analyses adjusting for additional covariates including %FVC, %DLco, BMI, ASMI, and serum albumin were performed and the C-index for predicting 3-year mortality computed. Univariate and multivariate binary logistic regression analyses were performed to examine associations between selected variables and respiratory-related hospitalization within 3 years. Correlations between serum transthyretin levels and clinical parameters were assessed using Spearman’s correlation coefficient. In a previous study [14], receiver operating characteristic curves for 1-year survival or respiratory-related hospitalization and the Youden index, which maximizes “sensitivity + specificity −1,” were used to determine an optimal cutoff of 22.6 mg/dL (sensitivity: 100%, specificity: 67.9%). In the present study, receiver operating characteristic analysis for 3-year survival yielded a similar cutoff of 23.5 mg/dL (sensitivity: 72%, specificity: 67%). Given the difference between these values and considering the clinical importance of 1-year outcomes, we adopted the previously reported cutoff of 22.6 mg/dL. Sensitivity analyses demonstrated consistent prognostic significance of low serum transthyretin across alternative cutoff values. The time to clinical outcomes was examined using the Kaplan–Meier method and compared using the log-rank test. Statistical significance was set at *p* < 0.05. Statistical analyses were performed using JMP statistical software (version 14; SAS Institute Japan Ltd., Tokyo, Japan) and EZR (Saitama Medical Center, Jichi Medical University, Saitama, Japan).

## 3. Results

### 3.1. Characteristics of the Patients

Three patients who used a wheelchair and, thus, could not perform gait speed measurement, seven who had insufficient data available, and six who declined to participate in the study were excluded. In total, 63 stable outpatients with IPF were enrolled in this study. Table 1 shows the characteristics of the patients. The mean age of the patients was 73.7 years, and the mean %FVC was 82.3%. In the study cohort, 38.1% (*n* = 24) of the patients had sarcopenia. The median serum transthyretin level was 24.0 [20.3–29.0] mg/dL. Nine patients were treated with corticosteroids. In eight patients, the corticosteroid dose was tapered after acute exacerbations, and another patient was on corticosteroids for a skin disease.

### 3.2. Clinical Outcome Survey

Of the 63 patients, 21 (33%) experienced respiratory-related hospitalizations during the 3-year follow-up period. Hospitalizations clearly attributable to heart failure were excluded from the definition of respiratory-related hospitalization. These events were attributed to acute exacerbations of IPF in 10 patients, infectious pneumonia in 7 patients, and pneumothorax in 1 patient. The remaining three cases involved respiratory failure of indeterminate cause, either alone or in combination with the aforementioned respiratory conditions. Overall, 18 patients (29%) died. In 12 patients, information on clinical outcomes was obtained through structured telephone interviews or written correspondence with patients or their families. To increase the validity of this information, respiratory-related hospitalizations and deaths were confirmed by reviewing hospital medical records whenever available. In cases managed at outside institutions, information was obtained from referring physicians or receiving hospitals. Outcome events were adjudicated by two pulmonologists based on predefined diagnostic criteria. As a result, no patients were lost to follow-up during the observation period.

### 3.3. Factors in the Cox Regression Analyses for All-Cause Mortality

Table 2 presents the hazard ratios (HRs) and 95% confidence intervals (CIs) from the Cox proportional hazards regression analyses for predicting all-cause mortality within 3 years. Given the limited number of outcome events, the multivariate model was restricted to the GAP index and serum transthyretin to minimize overfitting and maintain model stability. The GAP index (HR, 1.528; 95% CI, 1.157–2.019; *p* = 0.003) and serum transthyretin level (HR, 0.914; 95% CI, 0.848–0.985; *p* = 0.018) were identified as independent predictors of 3-year mortality. In contrast, after adjusting for the GAP index, ASMI and sarcopenia were not. In sensitivity analyses adjusting for additional covariates including %FVC, %DLco, BMI, ASMI, and serum albumin, the associations remained largely unchanged. The C-index for predicting 3-year mortality was 0.698 for serum transthyretin and 0.701 for the GAP index.

### 3.4. Factors in the Binary Logistic Regression Analyses for Respiratory-Related Hospitalization

Table 3 presents the odds ratios (ORs) and 95% CIs for respiratory-related hospitalization based on univariate and multivariate binary logistic regression analyses. Consistent with the mortality analysis and given the limited number of outcome events, we limited the multivariate model to the GAP index and serum transthyretin to reduce the risk of overfitting and preserve model robustness. The serum transthyretin level was identified anindependent predictor of the 3-year risk of respiratory-related hospitalization (OR, 0.901; 95% CI, 0.812–0.9990; *p* = 0.047).

### 3.5. Correlation of Serum Transthyretin with Clinical Parameters

Serum transthyretin levels were correlated with body mass index (r = 0.382, *p* = 0.002), GAP index (r = −0.322, *p* < 0.010), ASMI (r = 0.315, *p* = 0.012), and handgrip strength (r = 0.332, *p* = 0.008). Serum transthyretin levels did not correlate with age, ESM_CSA_, %FVC (r = 0.144, *p* = 0.262), %DL_CO_, gait speed, or serum surfactant protein D (SP-D) levels.

### 3.6. Kaplan–Meier Curves and Log-Rank Test

**Figure 1:**
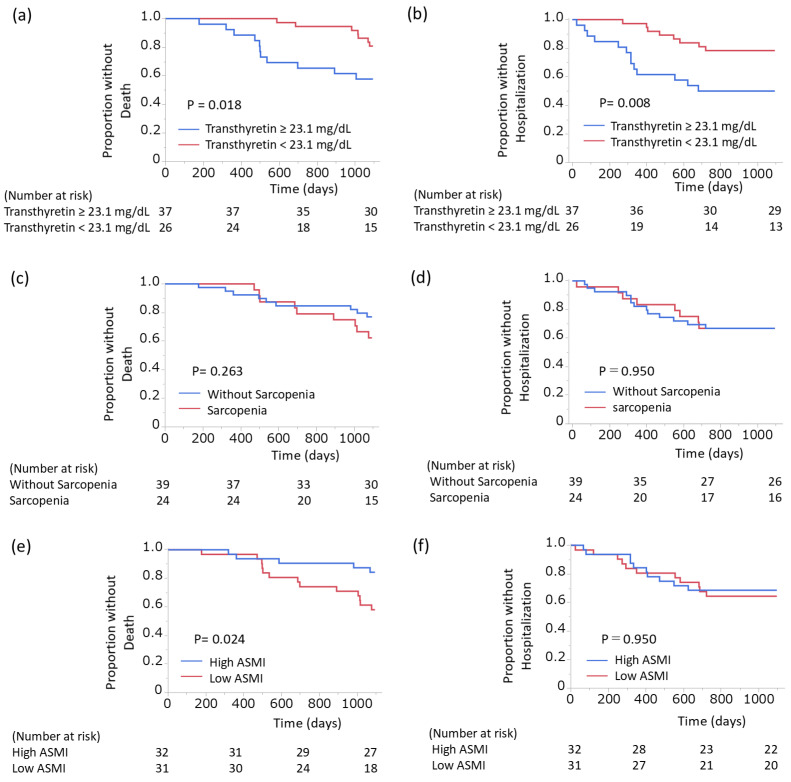
Kaplan–Meier curves for clinical outcomes according to serum transthyretin levels, sarcopenia status, and ASMI. Kaplan–Meier curves show death-free survival stratified by serum transthyretin levels (**a**), respiratory-related hospitalization-free survival stratified by serum transthyretin levels (**b**), death-free survival according to the presence or absence of sarcopenia (**c**), and respiratory-related hospitalization-free survival according to the presence or absence of sarcopenia (**d**). Death-free survival (**e**) and respiratory-related hospitalization-free survival (**f**) were also analyzed according to ASMI. Low ASMI was defined as <7.0 kg/m^2^ in men and <5.7 kg/m^2^ in women. The log-rank test showed significant differences in 3-year survival and respiratory-related hospitalization rates were observed between high and low serum transthyretin groups. Patients with low ASMI showed a significant difference in 3-year survival, but not in respiratory-related hospitalization rates. Abbreviations: ASMI, appendicular skeletal muscle mass index.

Kaplan–Meier curves are shown for death-free survival stratified by serum transthyretin levels (Figure 1a), respiratory-related hospitalization-free survival stratified by serum transthyretin levels (Figure 1b), death-free survival according to the presence or absence of sarcopenia (Figure 1c), and respiratory-related hospitalization-free survival according to the presence or absence of sarcopenia (Figure 1d). Low ASMI was associated with higher mortality (Figure 1e) but not with respiratory-related hospitalization (Figure 1f). Low ASMI was defined as <7.0 kg/m^2^ in men and <5.7 kg/m^2^ in women [16]. The log-rank test showed significant differences in 3-year survival and respiratory-related hospitalization rates were observed between high and low serum transthyretin groups. Sensitivity analyses demonstrated consistent prognostic significance of low serum transthyretin across alternative cutoff values.

### 3.7. Comparison of the Baseline Clinical Data Between the High and Low Serum Transthyretin Groups

Table 4 presents the baseline clinical characteristics of the high and low serum transthyretin groups. The high serum transthyretin group had a greater body mass index, and %DL_CO_, than the low serum transthyretin group. The GAP index was lower in the high serum transthyretin group than in the low serum transthyretin group. Further, the high serum transthyretin group had a lower prevalence of sarcopenia than the low serum transthyretin group.

## 4. Discussion

In this 3-year longitudinal study of patients with IPF, serum transthyretin levels were independently associated with both all-cause mortality and respiratory-related hospitalization, even after adjustment for the GAP index. Kaplan–Meier analyses demonstrated consistently poorer death-free and hospitalization-free survival among patients with low-er transthyretin levels. In sensitivity analyses, additional adjustment for covariates including %FVC, %DLco, BMI, and serum albumin did not alter the associations.

This study extends our previous findings [14] by demonstrating the independent prognostic relevance of serum transthyretin for long-term outcomes over a 3-year prospective follow-up and by comparing its predictive value with sarcopenia-related parameters. Our previous study [14] has suggested that serum transthyretin may be associated with short-term outcomes in patients with IPF. The present study extends these observations by prospectively following 63 stable Japanese outpatients for 3 years, simultaneously evaluating sarcopenia and serum transthyretin. This longer-term follow-up allows for a more comprehensive assessment of the prognostic role of transthyretin and its potential clinical utility in combination with established risk indices such as the GAP score.

Several mechanisms may explain the observed association between low serum transthyretin and adverse outcomes. First, transthyretin is a sensitive marker of protein–energy malnutrition. Nutritional deficiency impairs immune function and increases susceptibility to infections, which may precipitate acute exacerbations or respiratory deterioration in IPF. Second, transthyretin levels decrease in the setting of systemic inflammation [24,25]. Although fibrosis is the defining pathological feature of IPF, inflammatory and immune-mediated processes contribute to disease progression [26]. Thus, reduced serum transthyretin levels may reflect a combination of malnutrition and systemic inflammatory burden, both plausibly linked to mortality and hospitalization risk. Although serum transthyretin is widely used as a nutritional status marker, its serum levels can also be influenced by systemic inflammation, hepatic synthetic function, and acute-phase responses. In chronic inflammatory conditions such as IPF [26], transthyretin may therefore reflect a complex interplay between nutritional status, inflammatory burden, disease severity, and metabolic alterations.

Serum transthyretin levels were positively correlated with body mass index, ASMI, and handgrip strength, and negatively correlated with the GAP index. The correlations with ASMI (r = 0.315) and handgrip strength (r = 0.332), although statistically significant, were weak. Transthyretin levels did not correlate with age, ESM_CSA_, %FVC, %DL_CO_, gait speed, or serum SP-D levels. The absence of a significant correlation with %FVC indicates that pulmonary function decline does not necessarily parallel deterioration in nutritional status. Collectively, these findings suggest that serum transthyretin captures aspects of systemic nutritional and inflammatory status not fully reflected by muscle mass-based or pulmonary function-based measures, thereby providing complementary prognostic information.

The lack of a significant association between sarcopenia and long-term outcomes in the present analyses warrants further consideration. Sarcopenia diagnosed according to the AWGS 2019 criteria has been associated with impaired quality of life and depressive symptoms in IPF [18]. However, our results suggest that its independent predictive value for hard clinical endpoints such as death and respiratory-related hospitalization may be limited over a 3-year horizon. Given the weak but significant correlations between transthyretin and both ASMI and handgrip strength, serum transthyretin may capture broader systemic vulnerability beyond skeletal muscle quantity or strength alone. The absence of an independent association between sarcopenia and clinical outcomes may reflect differences in disease stage distribution, variability in assessment methods, or limited statistical power due to the small sample size. Although sarcopenia and low ASMI were not independent predictors in multivariate analyses, in unadjusted analyses, low ASMI was associated with 3-year survival. The missing association with respiratory-related hospitalization may reflect differences in disease stage distribution, assessment methods, or due to the limited sample size. Further, ASMI was assessed using bioelectrical impedance analysis, which may be influenced by factors such as hydration status and fluid distribution. In patients with chronic lung disease, these factors could introduce potential measurement bias; therefore, all findings related to muscle mass should be interpreted with caution.

The prevalence of sarcopenia in this cohort was 38.1%, higher than pooled estimates from recent meta-analyses [27]. Differences in age, disease severity, pulmonary function, clinical setting, and diagnostic methodology may explain this discrepancy. The absence of a significant association between sarcopenia and clinical outcomes in this study should be interpreted with caution. Functional parameters related to sarcopenia, such as muscle strength and physical performance, may change during follow-up. As no longitudinal reassessment of these parameters was performed, temporal changes in sarcopenia status could not be evaluated.

Low serum transthyretin levels may indicate systemic vulnerability related to malnutrition and inflammation in patients with IPF and be associated with lower tolerance to antifibrotic therapy. Serum transthyretin levels may help identify patients requiring closer nutritional monitoring and supportive care. In addition, as nutritional status influences lung transplantation outcomes, serum transthyretin may provide complementary information for risk stratification. Overall, serum transthyretin can be considered an adjunctive biomarker reflecting clinical vulnerability beyond conventional physiological indices.

Conventional measures such as body weight, body mass index, serum albumin, and body composition are clinically important. Since this small cohort study did not directly compare their prognostic performance with serum transthyretin, superiority cannot be assumed. However, given its short half-life and sensitivity to acute nutritional and inflammatory changes, transthyretin may provide complementary information beyond static measures. Future head-to-head, large cohort studies are needed to clarify its incremental prognostic value.

Low serum transthyretin is considered a dynamic marker that may improve with nutritional support or decreased inflammatory burden. Evidence from other chronic diseases suggests that transthyretin reflects potentially reversible systemic vulnerability related to malnutrition and frailty. However, whether such changes translate into improved outcomes in IPF remains unclear and warrants further investigation.

Weight loss is an established poor prognostic factor in IPF [11,12], and antifibrotic therapies frequently cause gastrointestinal adverse effects such as anorexia and nausea, potentially accelerating nutritional decline. Our findings raise the possibility that maintaining or improving nutritional status could favorably influence both survival and hospitalization risk. However, the optimal strategies for nutritional management in patients with IPF remain unclear. Future prospective interventional studies are needed to determine whether nutritional optimization or targeted metabolic interventions can improve transthyretin levels and, ultimately, clinical outcomes.

Although pulmonary function parameters and BMI may influence outcomes, the multivariate model was restricted to the GAP index and serum transthyretin to avoid overfitting due to the small sample size of this single-center study. Our approach balances statistical rigor with clinical interpretability in a small prospective cohort.

The C-index for predicting 3-year mortality was 0.698 for serum transthyretin and 0.701 for the GAP index. Their combination may improve prognostic discrimination.

We also performed sensitivity analyses for the cutoff value of serum transthyretin. Although alternative cutoffs for serum transthyretin did not affect the current results, multicenter, larger studies, may have a different optimal cutoff.

This study has several limitations that should be acknowledged. First, the single center design may limit the generalizability of the findings. Second, although the target sample size was determined based on the anticipated number of deaths, the observed event rates were lower than expected, possibly due to the widespread use of antifibrotic therapy. We acknowledge that some estimates have relatively wide CIs, reflecting the small size of this single-center study. This should be considered when interpreting the results. Although additional analyses incorporating key covariates were performed, residual confounding cannot be entirely excluded. Third, the proportion of patients with advanced disease (GAP stage III) was relatively small, which may have limited our ability to adequately assess the prognostic impact of serum transthyretin and sarcopenia in patients with more severe IPF. Fourth, as this study was conducted in a Japanese cohort, differences in ethnicity, body composition, and prevalence of sarcopenia may limit the generalizability of our findings to Western populations. Finally, although transthyretin is commonly used as a nutritional marker, it may also reflect systemic inflammation and overall disease burden.

## 5. Conclusions

Serum transthyretin was independently associated with both mortality and respiratory-related hospitalization over 3 years in patients with IPF, whereas sarcopenia defined according to the AWGS 2019 criteria [16] was not predictive of these outcomes. Although serum transthyretin showed statistically significant correlations with ASMI and handgrip strength, these associations were weak, suggesting that transthyretin provides complementary prognostic information beyond muscle mass-based assessments. Serum transthyretin may therefore serve as a complementary and clinically meaningful biomarker for risk stratification in IPF. However, given the observational nature of the study, serum transthyretin should be interpreted as a potential prognostic marker rather than a definitive risk stratification tool. The small number of outcome events (18 deaths and 21 respiratory-related hospitalizations) may have limited statistical power and increased the risk of model instability. Validation in larger multicenter cohorts is warranted.

## Figures and Tables

**Table 1:** Characteristics of the patients.

Variables	N = 63
Age, years	73.7 ± 7.9
Sex, male, n (%)	56 (88.9)
Body mass index, kg/m^2^	22.7 ± 3.3
ESM_CSA_, cm^2^	25.6 ± 6.7
Histological diagnosis, n (%)	19 (30.2)
Pulmonary function test	
FVC, % predicted	82.3 ± 15.6
DL_CO_, % predicted *	68.1 ± 18.8
Disease severity	
GAP index	3 [3, 4]
GAP stage (I/II/III)	38 (60.3%)/23 (36.5%)/2 (3.2%)
Physical assessment	
ASMI, kg/m^2^	6.8 ± 1.0
Gait speed, m/s	1.1 ± 0.3
Handgrip strength, kg	31.8 ± 10.1
Sarcopenia, n (%)	24 (38.1)
Blood examination	
Transthyretin level, mg/dL	24.0 [20.3–29.0]
SP-D level, ng/mL	215 ± 171
Treatment, n (%)	
Pirfenidone	27 (42.8)
Nintedanib	17 (26.9)
Corticosteroid	9 (14.2)

Data were presented as the means (±standard deviation), medians [interquartile range], or numbers (%). Abbreviations: ESM_CSA_, cross-sectional area of the erector spinae muscles at the level of the 12th thoracic vertebra; FVC, forced vital capacity; DL_CO_, diffusing capacity of the lung for carbon monoxide; GAP, Gender–Age–Physiology; ASMI, appendicular skeletal mass index; SP-D, surfactant protein D. * One patient could not undergo the DL_CO_ maneuver.

**Table 2:** Cox regression analyses for predicting 3-year mortality.

Predictors	HR	95% CI	*p*-Value
Univariate analysis			
Age	1.025	0.972–1.080	0.364
Sex, male	1.728	0.593–5.039	0.316
Body mass index, kg/m^2^	0.856	0.750–0.976	0.020
ESM_CSA_, cm^2^	1.000	0.999–1.000	0.506
FVC, % predicted	0.960	0.937–0.984	0.002
DL_CO_, % predicted *	0.949	0.925–0.973	<0.001
GAP index	1.700	1.296–2.230	<0.001
ASMI, kg/m^2^	0.606	0.405–0.905	0.014
Gait speed, m/s	0.523	0.129–2.121	0.364
Handgrip strength, kg	0.969	0.930–1.010	0.139
Sarcopenia	1.389	0.830–3.083	0.415
Transthyretin level, mg/dL	0.896	0.835–0.962	0.002
SP-D level, ng/mL	1.001	0.998–1.003	0.408
Antifibrotic drug	0.881	0.374–2.076	0.773
Corticosteroid	1.440	0.484–4.281	0.512
Multivariate analysis			
GAP index	1.528	1.157–2.019	0.003
Transthyretin level	0.914	0.848–0.985	0.018

Abbreviations: ESM_CSA_, cross-sectional area of the erector spinae muscles at the level of the 12th thoracic vertebra; FVC, forced vital capacity; DL_CO_, diffusing capacity of the lung for carbon monoxide; GAP, Gender–Age–Physiology; ASMI, appendicular skeletal mass index; SP-D, surfactant protein D. * One patient could not undergo the DL_CO_ maneuver.

**Table 3:** Binary logistic regression analyses for respiratory-related hospitalization within 3 years.

Predictors	OR	95% CI	*p*-Value
Univariate analysis			
Age	1.011	0.945–1.081	0.760
Sex, male	1.583	0.320–7.831	0.573
Body mass index, kg/m^2^	0.944	0.802–1.110	0.485
ESM_CSA_, cm^2^	1.000	0.999–1.001	0.494
FVC, % predicted	0.968	0.933–1.004	0.081
DL_CO_, % predicted *	0.949	0.915–0.984	0.005
GAP index	1.740	1.030–2.940	0.039
ASMI, kg/m^2^	0.735	0.424–1.273	0.272
Gait speed, m/s	0.714	0.097–5.233	0.741
Handgrip strength, kg	0.980	0.927–1.036	0.475
Sarcopenia	1.000	0.340–2.941	1.000
Transthyretin level, mg/dL	0.883	0.798–0.977	0.016
SP-D level, ng/mL	1.002	0.999–1.005	0.179
Antifibrotic drug	0.904	0.384–2.128	0.817
Corticosteroid	1.507	0.507–4.480	0.461
Multivariate analysis			
GAP index	1.480	0.847–2.585	0.168
Transthyretin level	0.901	0.812–0.999	0.047

Abbreviations: ESM_CSA_, cross-sectional area of the erector spinae muscles at the level of the 12th thoracic vertebra; FVC, forced vital capacity; DL_CO_, diffusing capacity of the lung for carbon monoxide; GAP, Gender–Age–Physiology; ASMI, appendicular skeletal mass index; SP-D, surfactant protein D. * One patient could not undergo the DL_CO_ maneuver.

**Table 4:** Comparisons of the baseline clinical data between the high and low serum transthyretin groups.

Variables	High Serum Transthyretin Group (≥22.6 mg/dL) n = 37	Low Serum Transthyretin Group (<22.6 mg/dL) n = 26	*p*-Value
Age	72.9 ± 8.4	74.8 ± 7.2	0.342
Sex, male, n (%)	34 (92%)	22 (85%)	0.438
Body mass index, kg/m^2^	23.6 ± 2.9	21.4 ± 3.5	0.007
ESM_CSA_, cm^2^	26.2 ± 6.5	27.6 ± 1.4	0.387
FVC, % predicted	84.3 ± 15.1	79.4 ± 16.0	0.220
DL_CO_, % predicted	72.2 ± 17.0	62.1 ± 20.0 *	0.036
GAP index	3 [2.5–4]	4 [3–5]	0.004
ASMI, kg/m^2^	7.0 ± 0.9	6.6 ± 1.1	0.060
Sarcopenia, n (%)	10 (27.0%)	14 (53.8%)	0.031
SP-D level, ng/mL	238 ± 204	182 ± 106	0.197
Antifibrotic drug	15 (41%)	16 (62%)	0.421
Corticosteroid	6 (16%)	3 (8%)	0.725

Abbreviations: ESMCSA, cross-sectional area of the erector spinae muscles at the level of the 12th thoracic vertebra; FVC, forced vital capacity; DLCO, diffusing capacity of the lung for carbon monoxide; GAP, Gender–Age–Physiology; ASMI, appendicular skeletal mass index; SP-D, surfactant protein D. * One patient could not undergo the DLCO maneuver.

## Data Availability

The data presented in this study are not publicly available due to privacy and ethical restrictions related to patient information. The data are available from the corresponding author upon reasonable request and with approval from the institutional review board.

## Abbreviations

IPF	idiopathic pulmonary fibrosis
GAP	Gender–Age–Physiology
ASMI	appendicular skeletal muscle mass index
CT	computed tomography
AWGS	Asian Working Group for Sarcopenia
DLCO	diffusing capacity of the lung for carbon monoxide
%FVC	percent predicted forced vital capacity
%DLCO	percent predicted diffusing capacity of the lung for carbon monoxide
ESMCSA	cross-sectional area of the erector spinae muscles at the level of the 12th thoracic vertebra
ESMs	erector spinae muscles
HR	hazard ratio
CI	confidence interval
SP-D	surfactant protein D
OR	odds ratio
